# A Tribute to Lewis Wolpert and His Ideas on the 50th Anniversary of the Publication of His Paper ‘Positional Information and the Spatial Pattern of Differentiation’. Evidence for a Timing Mechanism for Setting Up the Vertebrate Anterior-Posterior (A-P) Axis

**DOI:** 10.3390/ijms21072552

**Published:** 2020-04-07

**Authors:** Antony J. Durston

**Affiliations:** Institute of Biology, University of Leiden, Sylvius Laboratory, Sylviusweg 72, 2333 BE Leiden, The Netherlands; a.j.durston@gmail.com

**Keywords:** hox genes, limb development, main body axis, timing, time space translation

## Abstract

This article is a tribute to Lewis Wolpert and his ideas on the occasion of the recent 50th anniversary of the publication of his article ‘Positional Information and the Spatial Pattern of Differentiation’. This tribute relates to another one of his ideas: his early ‘Progress Zone’ timing model for limb development. Recent evidence is reviewed showing a mechanism sharing features with this model patterning the main body axis in early vertebrate development. This tribute celebrates the golden era of Developmental Biology.

## 1. Introduction: Lewis Wolpert and His Ideas

Lewis Wolpert is a giant in the arena of scientific discovery. Together with others, including Francis Crick, Sydney Brenner, Brian Goodwin, Janni Nüsslein-Volhaard, Ed Lewis, the Physicist Morrel Cohen and the Mathematicians René Thom and Christopher Zeeman, his ideas and discoveries in a period starting in the 1960′s helped establish that Developmental Biology is absolutely a key area of science. This was a period when ideas, rather than only technological developments, were recognised as the true currency of science. These pioneers’ original thinking and novel concepts reanimated the sleepy discipline of embryology. One sometimes fears that Developmental Biology’s pioneering spirit is now dead. We must ensure that it never dies. Original ideas and audacity lie at the root of any exciting science. They must be nurtured! They guarantee the future.

Wolpert’s key proposition, contained in [1], has been held to be that concentration gradients of morphogens acting on concentration thresholds can establish a developmental map in the embryo. This idea is undoubtedly of great importance and inspired a generation. It motivated many young scientists, including myself, and inspired us to become developmental biologists. However, Wolpert was also responsible for other important ideas and discoveries: to name just two: a very important early developmental timing model, his progress zone model for limb development [2,3,4,5,6], and his idea that an asymmetric molecule is the basis of left-right asymmetry (handedness) in the embryo [7]. The year 2019 was important for Lewis Wolpert. Not only was it the 50th anniversary of ‘Positional Information’, it was the 40th anniversary of his key publication reporting experimental evidence supporting his ‘progress zone’ model [3], although the original idea for this was already published six years earlier [2]. It was also his own 90th anniversary, and he was recently deservedly awarded the Royal Society’s Royal Medal. In this article, as a tribute to Lewis to celebrate these anniversaries, I point out evidence that a developmental timing mechanism, sharing properties laid bare by his ‘progress zone’ model is the prime candidate for patterning the vertebrate anterior-posterior (A-P) or rostro-caudal axis.

## 2. Wolpert’s ‘Progress Zone’ Model

**Figure 1:**
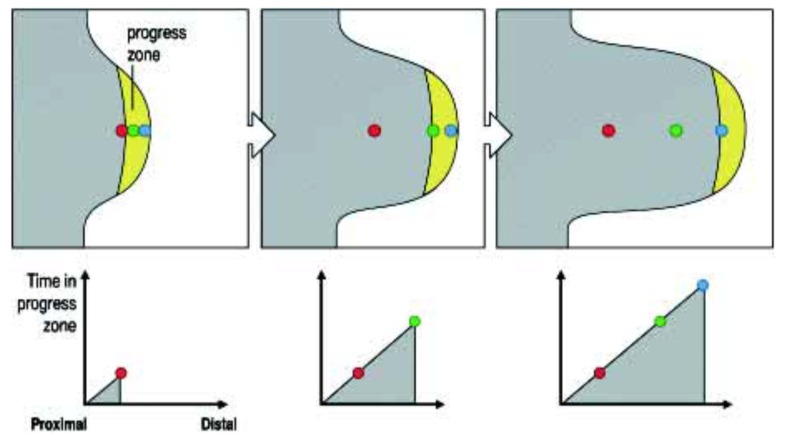
The Progress Zone Model. A cell’s proximodistal (PD) positional value may depend on the time it spends in the Progress Zone. Cells continually leave this zone at the tip of the limb under the apical ectodermal ridge. Cells that leave early form proximal structures while cells that leave last form the (distal) tips of the digits. Reprinted with kind permission from UPV/EHU Press from the source: Wolpert, L. (2002). The progress zone model for specifying positional information. *Int. J. Dev. Biol*. 46: 869–870 [5].

The Progress Zone (PZ) Model states that a 300 um deep zone of mesodermal cells in the distal tip of the developing limb is under the specific influence of a signal from the limb’s most distal (and external) apical feature: the apical ectodermal ridge (AER)—a known organiser. These mesodermal cells are growing and dividing and, while they are in the progress zone, their positional identity changes (it is dynamic and labile). The longer they or their progeny remain in this zone, the more distal their identity becomes. A timing mechanism is thus clearly indicated. Once these cells leave the PZ, their positional identity becomes fixed and can be manifested (see Figure 1).

This model was supported by the results of early X-irradiation of the developing limb tip [3]. It was found that this (which inhibits cell division and growth) causes defects in the proximal but not distal parts of the limb. This was interpreted to mean that a decrease in cell division and growth rate will cause cells to remain longer in the PZ so that their identity becomes more distal. There will be very few proximal cells that are able to make proximal structures.

This was a beautiful, simple and elegant model that clearly described a (or is it the only?) way in which a timing mechanism can pattern an axis. It was very influential in that it kicked up a storm of controversy [4,5,8,9,10]. This controversy inspired much new experimentation and brought on the limb field immensely. It soon became clear that the simplest form of the PZ model alone could not account for all of the data concerning proximodistal (PD) limb patterning. For example, it did not account for intercalary regeneration in grafts of proximal to distal limb bud sections [11]. That is unimportant. The PZ model and Lewis Wolpert’s pioneering spirit had already served their purpose. Lewis had continued the traditions of the pioneers. It is also clear that the PZ timing mechanism actually is a part of the proximodistal limb patterning mechanism [6,12,13]. 

Proximodistal limb patterning is clearly a complex process with multiple components, including signalling factors and a timer, which appear to be relevant at different phases. Wolpert’s progress zone ideas also seem relevant for patterning the main vertebrate anterior-posterior or rostro-caudal (A–P) body axis. The currently popular limb patterning mechanism has many components and features in common with the current proposed main A-P axis patterning mechanism. These include: some of the morphogens involved (*FGFs* and retinoids, *BMPs* and *Wnts), Hox* genes (*Hox a* and *d 9–13* in common), timing via trichostatin A sensitive histone acetylation (presumably because this leads to the collinear opening of Hox cluster chromatin), collinear *Hox-Hox* interactions, stage/PD position dependence for sensitivity to different morphogens. Wolpert’s progress zone model forms part of the current thinking. There is a candidate for the AER’s PZ signal: *FGF8* [14]. Other *FGF’s* (*FGF10, FGF4)* have also been proposed to be relevant [15,16]. The molecular nature of the PZ timer is currently under investigation [6,12,13,15,17,18,19]. This is further discussed below.

## 3. The Vertebrate A-P Axis Is Also Generated in A Timed Manner

Many findings show that the vertebrate A-P axis is generated in a timed manner, e.g., [19,20,21,22,23,24,25,26,27,28,29,30,31]. The current evidence points strongly to the idea that the axial timer is or involves *Hox* temporal collinearity (see below). It is interesting to ask whether this vertebrate axial timing mechanism has anything in common with Wolpert’s PZ model and/or with what has recently been established for the limb PD patterning mechanism.

## 4. What Is the Nature of the Vertebrate Axial Timing Mechanism?

**Figure 2:**
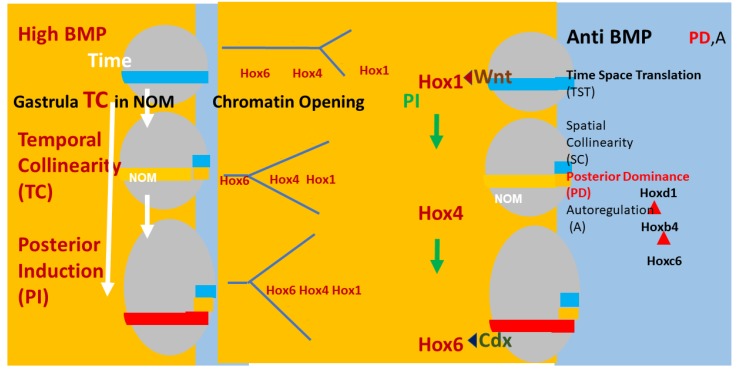
Timing, Axial patterning, and Time Space Translation. Left and right: (Xenopus) embryos (grey ovals) at 3 sequential stages in gastrulation. The non-organiser mesoderm (NOM) (horizontal coloured stripe) runs from ventral to near dorsal. I show some of the successive stages of temporally collinear Hox expression. First, blue stripe (Hoxd1 is the most posterior Hox gene expressed). Then, yellow stripe (Hoxb4 is the most posterior Hox gene expressed). Then, red stripe (Hoxc6 is the most posterior Hox gene expressed). These are three stages in the first part of the NOM temporally collinear (TC) Hox sequence. The yellow background shows that TC happens in the availability of a high BMP concentration, which is available in the ventrolateral part (V) of the embryo (as shown). Under these conditions, Collinear opening of chromatin and the Hox-Hox interaction posterior induction (PI) [36,37,38,39,40], which is necessary for TC, also occur as do Wnt and Cdx inputs into Hox1 genes and Hoxc6, respectively [41]. A thin segment at the right side of the embryo has a blue background (shown in detail for the embryos at the right side of the figure). This represents anti-BMP, which is available in the dorsal side of the embryo (D). Under these conditions, successive blocks of cells are frozen at each successive Hox code and these blocks stack up from early anterior to late posterior to make the A–P axis. This process involves making mesodermal and neural layers of spatially collinear tissue (separation not shown). It involves two late Hox–Hox interactions, Posterior Dominance, whereby posterior Hox genes inhibit function of and repress more anterior Hox genes and Autoregulation, whereby mesodermal Hox expression is copied over non cells autonomously from mesodermal to neural tissue [39,42].

There is evidence that A–P axial patterning in vertebrates is mediated by a timing mechanism and time space translation (TST) from gastrula stages onwards [19,29,30,32,33,34,35] (see Figure 2). 

To be brief: An integral core *Hox* collinearity mechanism applies for all *Hox* genes and employs collinear chromatin opening, collinear *Hox–Hox* interactions and the antagonism between *BMP* and anti-*BMP*. This, in turn, mediates a developmental timer, *Hox* temporal collinearity (TC) and time space translation (i.e., translation from *Hox* temporal collinearity to *Hox* spatial collinearity (TST), which is the basis for the axial pattern in the embryo). This main axis TC/TST machine matches Wolperts’s PZ model in that the operation of the timer depends on a diffusible signal. In the main axis, this is *BMP*, and there are also various external inputs (see below). In the PZ model, the signal is *FGF8*. The main axis TC/TST mechanism is also regulated by external inputs: morphogen signalling pathways, which act by being cofactors for expression of particular Hox genes, namely, in each case, the first *Hox* gene after a particular ‘decision point’ between two morphological domains on the body axis. Examples are: *Wnt*, acting at the anterior head-posterior head decision point; retinoids, acting at the same decision point; *FGF*, acting at the neck-thorax decision point; *Gdf11*, acting at the thorax-lumbar abdomen decision point. Please note that each of these external pathways acts only over a certain limited developmental period and only over a certain limited part of the A-P axis. The (extensive) evidence for this mechanism is given in three recent reviews [19,41,43]. Space prohibits repeating it here again. Please note that many of the features of this mechanism are also relevant for limb P-D patterning: namely, retinoids, *FGF*, *Hox* genes, chromatin histone acetylation, collinear opening of *Hox* cluster chromatin, *Hox–Hox* interactions, action of different morphogens at different stages. Both the limb workers and the A-P axis workers have made hypotheses about how all this applies in their particular field. We need to evaluate the possibility that there is a mechanism with common features that applies for both the limb P-D and main axis A-P mechanisms.

## 5. Do P-D Limb Patterning and A-P Body Axis Patterning Have A Common Basis? 

Table 1 shows the correspondence between components of limb P–D axis patterning and A–P body axis patterning. There are many parallels. This is perhaps not surprising considering that these are very well known developmental components.

**Figure 3:**
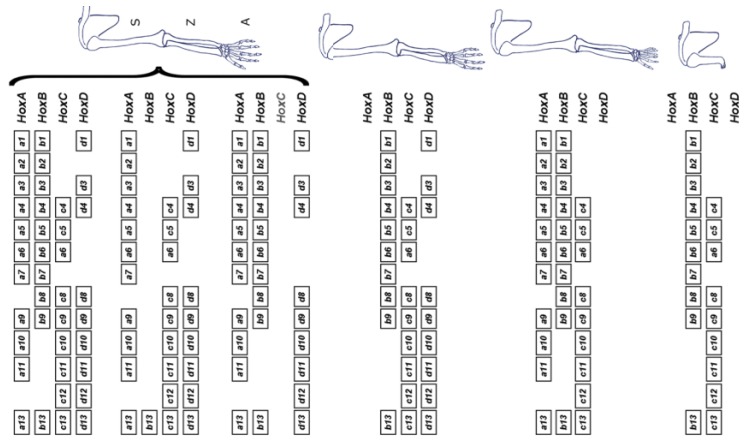
Irrelevance of the Hoxb and Hoxc clusters for limb development. On top, the full complement of *Hox* genes is shown (left), along with the associated wild-type morphology (right). The various schemes below illustrate full cluster deletions. Only the removal or either *Hoxa* or *Hoxd* leads to a detectable phenotype, which is not drastic and mostly affects the digital plate. However, the combined deletion of both *Hoxa* and *Hoxd* leads to an early arrest of limb growth, pointing to a large functional redundancy between these two clusters (S: stylopod, comprising the humerus and defining the upper arm; Z: zeugopod, comprising the radius and ulna and defining the lower arm; A: autopod, comprising the ensemble of carpus and digits). Reprinted from Elsevier, in keeping with the guidelines from the STM permissions association, from the source: Zakany J, Duboule D. The role of Hox genes during vertebrate limb development. *Curr. Opin. Genet. Dev*. 2007;17(4):359–366 [15].

I note the following points:

(1) Both the axial patterning mechanism and the limb patterning mechanism operate in the main axis of the structure concerned. In the body, this is the A-P axis; in the limb, the P-D axis, which are both patterned by *Hox* genes. However, the limb’s P-D axis is patterned together with the limb’s A-P axis, which is a minor axis in the limb.

(2) The axial patterning mechanism and the limb patterning mechanism are each regulated by a timer and time-space translation.

(3) Both show *Hox* temporal collinearity that precedes and apparently generates *Hox* spatial collinearity, which generates the body’s A-P axial pattern and the main (P-D) axis pattern and structure in the limb. This is called time space translation (TST). TST mediates patterning and growth in the body axis and, although the evidence is somewhat less complete, it clearly does the same in the limb (see below). The evidence is, thus, clear that *Hox* temporal collinearity is the developmental timer both in the main A-P axis of the embryo and in the P-D axis of the limb.

(4) The *Hox* timer (TC) in the body axis is *BMP* dependent. The diffusible signal *BMP* (produced in the NOM mesoderm/primitive streak, where the timer runs) allows/generates a timed sequence of nascent positional values. TST and stopping of the timer are induced by timed anti-*BMP*. An A-P sequence of positional values are stabilised at an early to late sequence of times. In the limb, *FGF8* [14] (and perhaps other *FGF’s*) seems to be the AER’s signal that corresponds to *BMP*, which enables the timer and, presumably, *FGF* antagonism or subthreshold *FGF* would stabilise positional values (as with anti *BMP*). The limb story is, however, complex. Unlike in the main body axis, different *Hox* clusters are very differently regulated. Only the *Hoxa* and *Hoxd* clusters are relevant during limb development. Deletion of the *Hoxb* and *Hoxc* clusters does not affect it [15] (see Figure 3).

**Figure 4:**
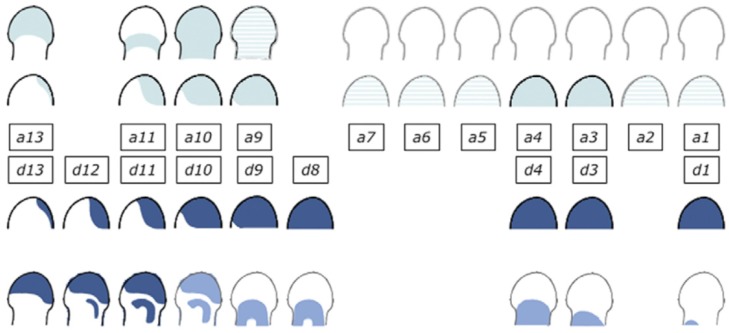
*Hoxa* and *Hoxd* expression in the developing limb. Two phases of expression of *Hoxa* and *Hoxd* genes. During phase 1, genes are activated sequentially in both clusters, following the same general collinear strategy, with ‘posterior’ genes (e.g., group 13) transcribed in progressively more posterior cells of the limb buds. During the second (late) phase, *Hoxa* and *Hoxd* patterns are still quite comparable, but with obvious differences, suggesting that different regulations are now implemented. Reprinted from Elsevier, in keeping with the guidelines from the STM permissions association, from the source: Zakany J, Duboule D. The role of Hox genes during vertebrate limb development. Curr Opin Genet Dev. 2007;17(4):359–366 [15].

The *Hoxd* cluster is best studied. Investigation of its function shows clearly that the *Hoxd* cluster regulates limb P-D as well as A-P development. I should mention here that a great deal of the excellent work that has been done on the role and regulation of *Hoxd* genes in limb development has been by D. Duboule and his colleagues, e.g., [15,44]. Regulation of the *Hoxd* genes is complex in that it shows two phases. In phase 1, during earlier development of the limb, *Hoxd9-13* are expressed sequentially in time in nested ‘Russian doll’ patterns, centred in the distal-posterior part of the limb bud. More 3′*Hox* genes are expressed sequentially too, but their expression shows no localisation. It is clear that these patterns are regulated by A-P as well as P-D signals. The expression patterns are very similar to the expanding Russian doll patterns seen in the developing mouse main axis when TC generates SC. I suggest that the same is happening here too (Figure 4).

The posterior-distal location of the TC focus indicates that as well as being regulated by the limb’s P-D factor *FGF8*, this is regulated by the limb specific A-P factor, sonic hedgehog (*Shh*). Both of these factors are presumably required for temporal collinearity in this phase. In a second phase, during later development of the limb’s autopod (hand plate), the patterns remain posteriorly distal for *Hoxd* genes but become purely distal for *Hoxa* genes. There are, thus, two diffusible factors (*FGF8*/ other relevant *FGF* and *Shh*) potentially involved in limb *Hox* collinearity. Possibly, only *FGF8* remains relevant for later expression of *Hoxa* genes. However, there is also an indirect role for *BMP*-anti *BMP* too. This regulates the viability and availability of the AER and, therefore, presumably regulates the *FGF* signal emitted by the AER that permits the limb timer to run in the progress zone [45]. This signal is, thus, repressed by *BMP* and enhanced by anti-*BMP*. These *BMP* dependent functions in the limb are clearly different from those in the body axis.

(5) 5′ *Hox a* and *d* genes are clearly centrally involved in limb development (see above). Their importance has been examined in the forelimb by combined loss of function for both paralogues, leading to combined *Hox a* and *d* loss of function. Loss of function for the *Hox 9* or *10* paralogues causes defects at sequential levels in the stylopod (presumptive upper arm) [15,46,47,48], loss of function of *Hox11* causes defects in the zeugopod (presumptive forearm) [15,47,49], loss of function for *Hox 12* or *13* paralogues modifies or deletes the autopod (presumptive hand and wrist) [15,47,50,51]. The developmental timer (*Hox* temporal collinearity), thus, operates sequentially throughout early and mid-limb development (the stages when the stylopod, zeugopod and autopod are specified sequentially). *Hox 9-10* are involved sequentially in specifying the stylopod, *Hox 11* in specifying the zeugopod and *Hox 13* in specifying the autopod [15]. In addition to being regulated by *FGF’s* and *Shh*, the *Hox* genes regulate these factors themselves. *Hox 9* and *10* upregulate *FGF10* and *Shh. Hox 13* downregulates *FGF10*. This feedback presumably relates to the obvious connection between limb A-P and P-D patterning. These data are all consistent with Hox temporal collinearity mediating Wolpert’s PZ timer for proximodistal limb patterning. I note that the timer’s action during earlier limb development is somewhat masked by the fact that the timer’s necessary distal diffusible signal, *FGF8*, is antagonised by a proximal signal, retinoic acid [12,13,17]. The period of temporal collinearity operation is, thus, potentially the same period of operation as for Wolpert’s progress zone timer and the idea that *Hox* temporal collinearity mediates this timer has been proposed previously [12]. The reasoning here supports this view. An alternative idea has been that the timer is cell cycle based [2,13]. 

(6) It was at one point proposed previously that limb P-D patterning is mediated by morphogens alone, without any intrinsic (timing) mechanism [52]. It actually seems most likely that these diffusible morphogens interact with an endogenous timing mechanism to pattern the limb’s P-D axis [12,13,17]. Some of the morphogen signals involved in patterning the main body axis have their best characterised effects early and anterior in the axis. Too early and too anterior to be relevant for limb patterning. However, the same morphogens have multiple effects including (so far, less well characterised) effects on the posterior axis. We can, for example, expect retinoids to have a function in the abdominal part of the axis or at the abdomen/tail boundary, a position where they can truncate the axis or induce limbs in place of a tail [53,54]. Some of these more posterior functions may well relate to morphogen functions in the limb.

## 6. Questions for the Future

(1) Does *Hox* Temporal Collinearity Actually Exist? Two recent publications question whether *Hox* temporal collinearity actually exists [55,56]. I have presented the arguments that it does and that it is of central importance [57,58]. This question needs to be settled urgently and definitively.

(2) Can These Insights Be Used in Connection with Stem Cells? The mechanism above is an important part of the program generating the diversity of cell types and organs that make an animal. The investigation by Faiella C.S. already demonstrated a long time ago that part of this mechanism can operate in a pluripotent cell line [36]. With the diversity of ES cells now available, it will be important to determine whether this *Hox* mechanism can be used to generate and further new stem cell applications. It should also have perspectives for in vitro organoid culture. 

(3) What Is the Nature of the Timer? *Hox* temporal collinearity drives the timing and spatial sequence of A-P axial patterning and probably also of limb PD patterning. However, is temporal collinearity itself the driver or is it in turn driven by something else? Is it itself precise enough to drive a developmental program? This is an important question. There is a second time-space translation mechanism active in the early embryo as well as in the developing limb [59], in the same tissues and with the same timing as Hox temporal collinearity. This mechanism is active in NOM mesoderm (anamniotes) or primitive streak (amniotes) and later in the presomitic mesoderm. It is active during gastrulation (chicken and *Xenopus* [60,61] but is also already known to be active by the zebrafish’s earlier blastula stage (when the head starts its specification) [62]. This mechanism (the somitogenesis clock) is presumably precise because it is based on (many ticks of) a relatively high frequency stable oscillator (the limit cycle characteristics of which should ensure stability and which can deliver precision if elapsed cycles are counted) and it is known to be able to drive *Hox* temporal collinearity [61]. Temporal collinearity, however, also feeds back to drive it [38]. These two TST mechanisms are, thus, clearly connected. What drives what?

(4) What Is the Nature of *Hox-Hox* Interactions? The mechanism for generating *Hox* temporal collinearity and translating it to a spatially collinear pattern is complex. Multiple collinear *Hox–Hox* interactions appear to be involved. Temporal collinearity appears to require an interaction where each *Hox* gene induces its posterior neighbours, Posterior Induction (PI). PI was deduced from cascade phenotypes in *Xenopus* and in NT2/D1 cells, which were all obtained using either ectopic expression (gain of function) or antisense technology (morpholinos or regular antisense oligonucleotides; loss of function) [36,37,38,39,40]. These phenotypes appeared very specific and not artefactual because each generated expression of a very specific sequence of *Hox* genes either starting with the treated *Hox* gene and running towards late/posterior (gain of function) or running from anterior/early up to and stopping with the marker before the treated *Hox* gene (loss of function). However, it would be very instructive to see what kinds of *Hox* expression phenotypes other standard gene manipulation approaches (like ectopic expression in mouse, homologous recombination in mouse, CRISPR) give. This is so far largely unknown. There have been investigations using ectopic expression and knockouts as well as genetic rearrangements in mouse that provide information about one interaction: posterior prevalence/ posterior dominance (PP/PD) [15,44]. These are informative, but much more needs to be done.

(5) What Are the Roles of Morphogens? There are various morphogens that are thought to be involved in setting up the main body’s A–P axis and the limb’s P-D axis. Their roles in relation to the timing mechanism considered here have been discussed above and elsewhere [41,43]. However, this aspect deserves further attention. There is lots more to be done.

(6) Do the limb P-D and body axis A-P timing mechanism and TST have a common molecular basis? This complex problem needs to be solved (see above).

## 7. Hearty Congratulations to Lewis on These Important Anniversaries

May creative science in general, Developmental Biology in particular and Lewis himself thrive!

## Figures and Tables

**Table 1:** Common features, between main body A-P axis and limb P-D axis development.

Characteristic	A-P Main Axis	PD-AP Limb
**Morphogens**		
*FGF*	**+ (Hox6)**	**+ (AER)**
*Retinoid*	**+ (Hox1)**	**+ (proximal)**
*Wnt*	**+**	**+**
*BMP*	**+ Axis connection D-V-A-P**	**+ Axis connection D-V-P-D**
**Chromatin**		
**Histone acetylation**	**+**	**+**
**Hox genes**	**+ (1–13) TC,SC.**	**+ (9–13) TC, SC.**
**Timer**	**+**	**+**
**Stage dependence**	**Early anterior**	**Early proximal**
